# Epigenomic Landscape of Human Cumulus Cells in Premature Ovarian Insufficiency Using Single‐Base Resolution Methylome and Hydroxymethylome

**DOI:** 10.1111/jcmm.70284

**Published:** 2024-12-20

**Authors:** Wenhao Shi, Dongyang Wang, Xia Xue, Sen Qiao, Wei Zhang, Juanzi Shi, Chen Huang

**Affiliations:** ^1^ Department of Cell Biology and Genetics, School of Basic Medical Sciences Xi'an Jiaotong University Health Science Center Xi'an Shaanxi China; ^2^ The Assisted Reproduction Center Northwest Women's and Children's Hospital Xi'an Shaanxi China; ^3^ Internal Medicine Department Shaanxi Provincial People's Hospital Xi'an Shaanxi China

**Keywords:** DNA hydroxymethylation, DNA methylation, epigenetic, ovarian function, premature ovarian insufficiency

## Abstract

Premature ovarian insufficiency (POI) has recently been reported to be linked with epigenetic changes. Previous studies have focused on the regulation of individual genes associated with ovarian function through single‐gene epigenetic variations; however, there is a deficiency in the comprehensive comprehension of the epigenetic profile for POI. Therefore, we conducted a multi‐omics study integrating methylation, hydroxymethylation and transcriptome sequencing analyses in cumulus cells from women with POI and their matched controls. Our data revealed significant global increases in methylation and hydroxymethylation levels in POI patients. We observed a predominance of hypermethylated and hyperhydroxymethylated regions across the genome, with methylation in gene bodies negatively correlating with gene expression, especially in promoter regions. Subsequent experimental validation was performed to confirm the involvement of candidate genes (EGR1, EGR2 and DLX5) in ovarian steroid hormone synthesis. Interestingly, our findings indicate that these epigenetic modifications are associated with genes implicated in POI, ovarian function and the epigenetic age clock. This comprehensive epigenetic profile underscores the potential for identifying novel biomarkers and therapeutic targets for POI by unravelling the complex interplay between DNA epigenetics and ovarian function.

## 1 Introduction

Premature ovarian insufficiency (POI) affects approximately 3.7% of women under 40 [1], leading to decreased ovarian function, higher gonadotropin and lower estradiol levels and even amenorrhea [2]. Despite its prevalence, the molecular causes of POI remain largely unknown, with most cases labelled idiopathic after assessment [3]. While genetic mutations are thought to play a significant role, only 23.5% of POI cases can be linked to genetic variations according to a large‐scale whole exome sequencing study [4]. Increasingly, research points to a combination of physiological, psychological and environmental factors as potential triggers for POI [5, 6]. These factors may induce epigenetic changes, such as DNA methylation and hydroxymethylation, altering gene expression without modifying the DNA sequence itself [7].

Epigenetic mechanisms, including DNA methylation and hydroxymethylation, are crucial in regulating organ development and function across the lifespan, playing a significant role in aging [8]. The ovary, often one of the first organs to show signs of aging, may undergo epigenetic changes that contribute to its decline. POI is a condition associated with early ovarian aging [9], and emerging evidence suggests that DNA methylation could be involved in its development [7, 10]. This leads to the hypothesis that epigenetic alterations in the ovary may underlie the pathogenesis of POI, distinguishing affected individuals from those with normal ovarian function.

## 2 Materials and Methods

### 2.1 Patients

Patients undergoing in vitro fertilisation (IVF) at the Northwest Women's and Children's Hospital's Assisted Reproductive Technology Center were selected for this study. POI patients were under 40, had high FSH levels (> 20 mIU/mL), antral follicle counts (AFC) below 5, lacked high‐grade Day 3 embryos and had previous IVF failures. The control group (Ctrl) was first‐time IVF patients without POI, had male or tubal factor infertility and showed good prognosis with ≥ 10 oocytes and ≥ 5 high‐grade Day 3 embryos, closely age‐matched (± 1 year) to the POI group. Exclusion criteria included chromosomal abnormalities, abnormal BMI (< 18.5 or > 24 kg/m^2^), history of ovarian surgery, chemotherapy, radiotherapy, autoimmune disorders, hypertension, diabetes, tuberculosis, or conditions affecting oocyte/embryo development. Baseline characteristics of both groups are detailed in Table S1.

### 2.2 IVF Protocols and Sample Collection

Patients underwent a standard gonadotropin‐releasing hormone antagonist protocol for controlled ovarian stimulation. Cumulus oocyte complexes (COCs) were retrieved in G‐IVF PLUS TM culture medium during oocyte retrieval. The COCs were denuded through mechanical methods and the use of hyaluronidase (40 IU) to eliminate cumulus cells. Subsequently, the cumulus cells were washed, centrifuged and harvested.

### 2.3 Whole‐Genome Bisulfite Sequencing (WGBS) and Hydroxymethylome Sequencing

WGBS utilises the property of bisulfite to convert unmethylated cytosines (C) into thymine (T). After treating the genome with bisulfite, sequencing is performed as previously described [11]. APOBEC‐Coupled Epigenetic Sequencing (ACE‐Seq) was introduced, and this is a bisulfite‐free method for localising 5‐hydroxymethylcytosine (5hmC) at single base resolution [12]. The sequencing quality data for the six samples can be found in Table S2.

### 2.4 Validation of Target Gene Methylation Levels

The amplicon bisulfite sequencing (AmpBS) technique was used to validate the methylation levels of target genes. Specific primers were designed for the target fragments on the genome, followed by PCR amplification and recovery. Subsequently, bisulfite‐treated libraries were constructed for sequencing, allowing for accurate detection of DNA methylation in the target fragments.

### 2.5 Bioinformatical Analysis

Low quality bases and adapter sequences were trimmed by using trimmomatic (v0.38) [13]. Then the clean data were mapped to reference hg19 using BSMAP software version 2.90 [14]. Methylation ratios were extracted from BSMAP output (SAM) using a Python script (methratio.py), which is distributed with the BSMAP package. Differentially methylated cytosines/Differentially hydroxymethylated cytosines were detected using MethylKit (v1.20) [15] in de‐novo mode among CpG sites with at least 5× coverage. Differentially methylated regions (DMRs)/differentially hydroxymethylated regions (DhMRs) were detected using metilene [16] in de‐novo mode among CpG sites with at least 5× coverage. An integrative multi‐omics analysis on single samples was conducted, merging whole‐genome methylation/hydroxymethylation with transcriptome data. Correlation density plots showed the link between gene expression and methylation/hydroxymethylation across promoter and gene body regions, using shades of blue to represent methylation levels. The correlation coefficient (R) revealed the direction of correlation, with positive and negative values indicating direct and inverse relationships, respectively.

### 2.6 Transcriptome Sequencing and Analysis

TPM (transcripts per kilobase of exon model per million mapped reads) values were calculated by StringTie (v2.1.7) [17] and were used to estimate the expressed values. Differentially expressed genes (DEGs) were obtained using DEseq2 (v1.34.0) [18] with a *p* value cutoff < 0.05 and an absolute log_2_(fold‐change) of > 1. KEGG and GO enrichment analysis was performed on the clusterProfiler R package (v4.2.2) [19]. Reverse transcription and quantitative PCR (RT‐qPCR) was performed to confirm the results obtained with transcriptome analysis. The primers used are listed in Table S3. Gene expression was analysed using the 2^−ΔΔCq^ method, and β‐actin (ACTB) was chosen as the reference.

### 2.7 Preliminary Functional Experiments of Cells In Vitro

Human ovarian granulosa KGN cells (Pricella Life Science&Technology, China) were cultured and treated with 100 ng/mL FSH (MedChemExpress, China) for various durations. For gene manipulation, KGN cells were infected with lentivirus carrying either the DLX5 gene (NM_005221.6) or shRNA against DLX5 (5′‐caaaccaaagaaagttcgtaa‐3′), along with a puromycin resistance gene, sourced from Genecarer Bio. Post‐infection, cells were selected with puromycin (Deeyee, China) to obtain DLX5 overexpressed or knocked down cells, verified by RT‐PCR. To assess hormonal changes, these modified cells and controls were treated with testosterone (Solarbio Life Sciences, China) and forskolin (MedChemExpress, China) in a low serum medium. Estradiol (E2) and progesterone (P) levels in the culture medium were measured using specific chemiluminescence enzyme immunoassay (CLIA) kits from Beckman Coulter.

### 2.8 Methylation Levels Among POI Causative Genes, Ovarian Function Genes and Epigenetic Age Clock

We analysed methylation differences between the POI group and controls using heatmaps for 101 POI‐related and 703 ovarian function genes (Table S4), based on whole‐exome sequencing data from 1030 POI patients [4]. Additionally, we compared methylation at epigenetic age clock CpG sites between both groups using another heatmap, referencing CpG sites from established clock models (Table S5) [20, 21, 22, 23, 24, 25].

### 2.9 Statistical Analysis

Data were presented as mean ± standard deviation, analysed using GraphPad Prism 9. Differences between two groups were assessed with the Student's *t*‐test, and among multiple groups with one‐way ANOVA and Tukey's test. A *p*‐value < 0.05 was considered statistically significant.

## 3 Results

### 3.1 Sequencing Strategy and Quality Control

We performed WGBS‐seq, ACE‐seq and RNA‐seq on cumulus cell samples from three POI patients and three matched controls, as detailed in Table S1. WGBS‐seq and ACE‐seq yielded 575 to 630 million raw reads for six DNA samples (Table S2), with high‐quality DNA ensuring effective sequencing depth (Figure S1). Post‐quality control, over 80% of reads were successfully aligned to the reference genome. High conversion rates were noted in WGBS (98.41%–98.61%) and ACE libraries (96.34%–98.44%), indicating effective bisulfite and APOBEC3A enzyme treatment, respectively. We analysed 28.69–29.05 million CpG sites for hydroxymethylation patterns, with Circos plots visualising methylation/hydroxymethylation distribution across chromosomes for both groups (Figure S2).

### 3.2 Global 5mC Level and Distribution

**FIGURE 1:**
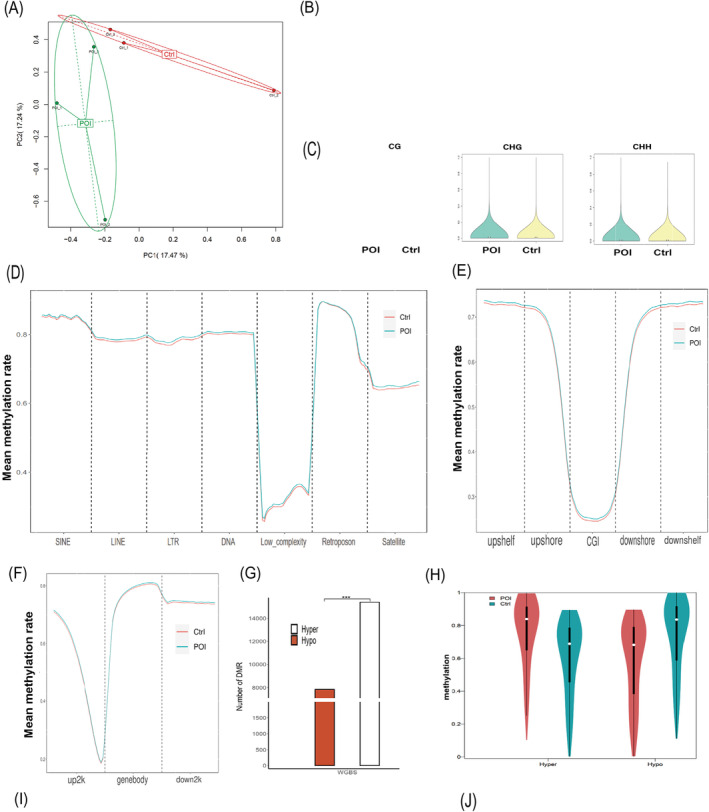
Comprehensive analysis of genome‐wide DNA methylation and hydroxymethylation status in cumulus cells between POI and control women. (A) Principal Component Analysis (PCA) reflects the main features of the methylation profiles. (B) The global methylation average rate of CG/CHG/CHH. (C) the violin plot to display the distribution density of whole‐genome methylation levels between groups. (D–F) Distribution of methylation in genomic repeat elements (SINEs, LINEs, LTRs, Low_complexity, Retroposon, Satellite and Simple repeats), in human genomic regions (CpG islands, shelf and shore) and in 2000 bp upstream of the transcription start site (up2k), gene body (genebody), and 2000 bp downstream of the transcription end site (down2k). (G) The number of differentially methylated region (DMR) with Hyper and Hypo type. (H) The violin chart was used to display the distribution density of DMR with Hyper and Hypo type across different intervals of the entire genome in each group. (I, J) The DMR distribution on chromosomes and on gene elements (3utr, 5utr, exon, intergenic, intron, promoter). WGBS, Whole‐genome bisulfite sequencing.

Principal component analysis (PCA) showed clear differences in methylation profiles between the POI and controls (Figure 1A). Globally, the POI group exhibited higher average levels of 5mC at CG/CHG/CHH sites (73.95%/1.37%/1.29%) compared to controls (73.40%/1.25%/1.18%) (Figure 1B). Although the overall methylation patterns for CG, CHG and CHH sites were similar across both groups (Figure 1C), detailed analysis revealed that 5mC levels were significantly elevated in POI patients across various genomic regions, including repeat elements like SINEs, LINEs, LTRs and CpG islands, shelves and shores, as well as gene structural elements (up2k, gene body, down2k) compared to controls (Figure 1D–F).

### 3.3 Global 5hmC Level and Distribution

**FIGURE 2:**
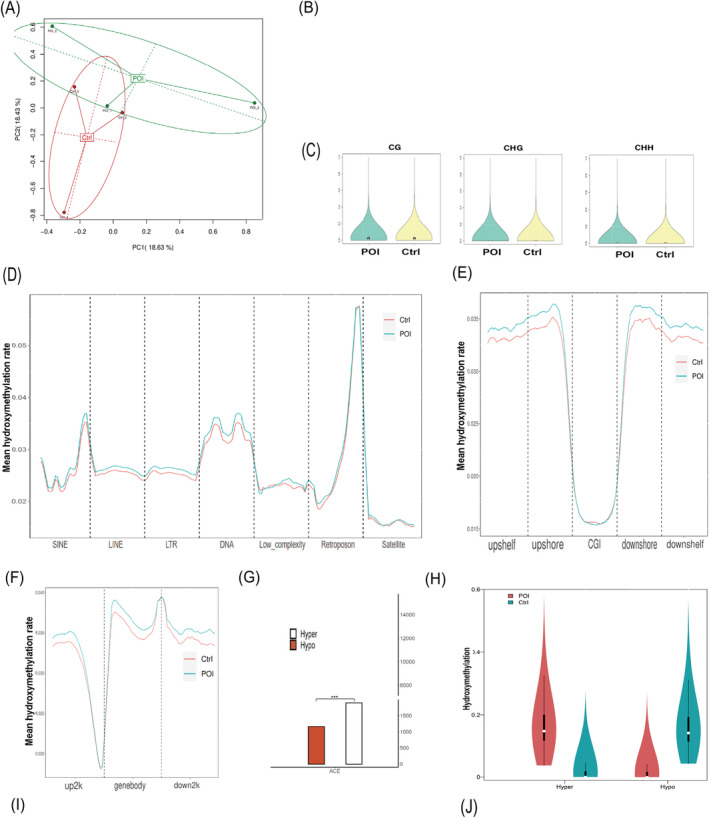
Comprehensive analysis of genome‐wide DNA hydroxymethylation status in cumulus cells between POI and control women. (A) Principal Component Analysis (PCA) reflects the main features of the hydroxymethylation profiles. (B) The global hydroxymethylation average rate of CG/CHG/CHH. (C) The violin plot to display the distribution density of whole‐genome hydroxymethylation levels. (D–F) Distribution of hydroxymethylation in genomic repeat elements (SINEs, LINEs, LTRs, Low_complexity, Retroposon, Satellite and Simple repeats), in human genomic regions (CpG islands, shelf and shore) and in 2000 bp upstream of the transcription start site (up2k), gene body (genebody), and 2000 bp downstream of the transcription end site (down2k). (G) The number of differentially hydroxymethylated regions (DhMRs) with Hyper and Hypo type. (H) The violin chart was used to display the distribution density of DhMR with Hyper and Hypo type across different intervals of the entire genome in each group. (I, J) The DhMR distribution on chromosomes and on gene elements (3utr, 5utr, exon, intergenic, intron, promoter). ACE, APOBEC‐Coupled Epigenetic Sequencing.

PCA also showed clear differences in hydroxymethylation profiles between two groups (Figure 2A). In the POI group, the average global levels of 5‐hydroxymethylcytosine (5hmC) at CpG sites were slightly higher (2.77%) compared to the control group (2.68%), while at CHG/CHH sites, the levels were slightly lower in the POI group (0.87%/0.88%) than in controls (0.89%/0.89%) (Figure 2B). Hydroxymethylation was distributed across all chromosomes (Figure S2), with higher levels of 5hmC in genomic repeat elements, CpG islands, shelves, shores and gene structural elements (up2k, gene body, down2k) in POI patients versus controls (Figure 2D–F), indicating a similar pattern of hydroxymethylation across genomic regions and chromosomes between the two groups.

### 3.4 The DMRs and DhMRs

Between the POI and control groups, we identified 23,234 DMRs comprising 15,389 hypermethylated and 7845 hypomethylated regions, along with 3058 DhMRs, with 1892 being hyperhydroxymethylated and 1166 hypohydroxymethylated. Circos diagrams illustrated the genome‐wide distribution of these DMRs/DhMRs (Figure S3). Notably, hypermethylation/hyperhydroxymethylation predominated over hypomethylation/hypohydroxymethylation in our data (Figures 1 and 2G). Both DMRs and DhMRs showed a similar distribution density across different genomic regions (Figures 1 and 2H). Chromosomal analysis revealed that autosomes had more hypermethylated DMRs than hypomethylated ones, a trend not seen in sex chromosomes or mitochondria (Figures 1 and 2I). We also charted the gene structural elements associated with these methylation/hypohydroxymethylation changes (Figures 1 and 2J).

### 3.5 RNA Sequencing Data Analysis

**FIGURE 3:**
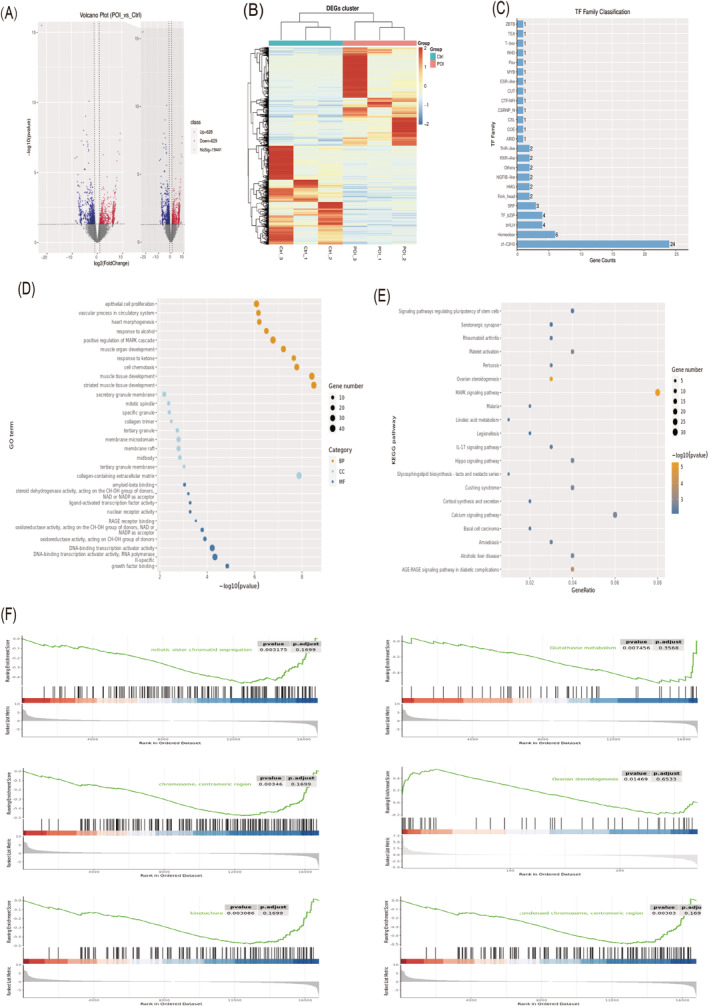
Analysis of differentially expressed genes in the transcriptome. (A) The volcano plot of differential expression genes. (B) The clustering heatmap analysis on the differentially expressed genes between all groups. (C) Transcription factors (TFs) family classification. (D, E) GO and KEGG pathway analysis for differentially expressed gene. (F)The Gene Set Enrichment Analysis (GSEA) revealed ovarian‐related pathways among the top 10 pathways.

Among the identified DEGs (*n* = 1257), 628 genes were upregulated and 629 genes were downregulated (Figure 3A,B). We explored the distribution of transcription factor families in DEGs (Figure 3C). GO and KEGG pathway analysis was performed for DEGs (Figure 3D,E; Figure S4). In the top 10 pathway analysis of gene set enrichment analysis (GSEA, Figure 3F), the POI group showed enriched and downregulated gene expression in these ovarian function‐related pathways (mitotic sister chromatid segregation; chromosome, centromeric region; kinetochore; condensed chromosome, centromeric region; glutathione metabolism; ovarian steroidogenesis) compared to the control group.

### 3.6 Experimental Validation to Detect DNA Methylation, mRNA and Biological Function Changes

**FIGURE 4:**
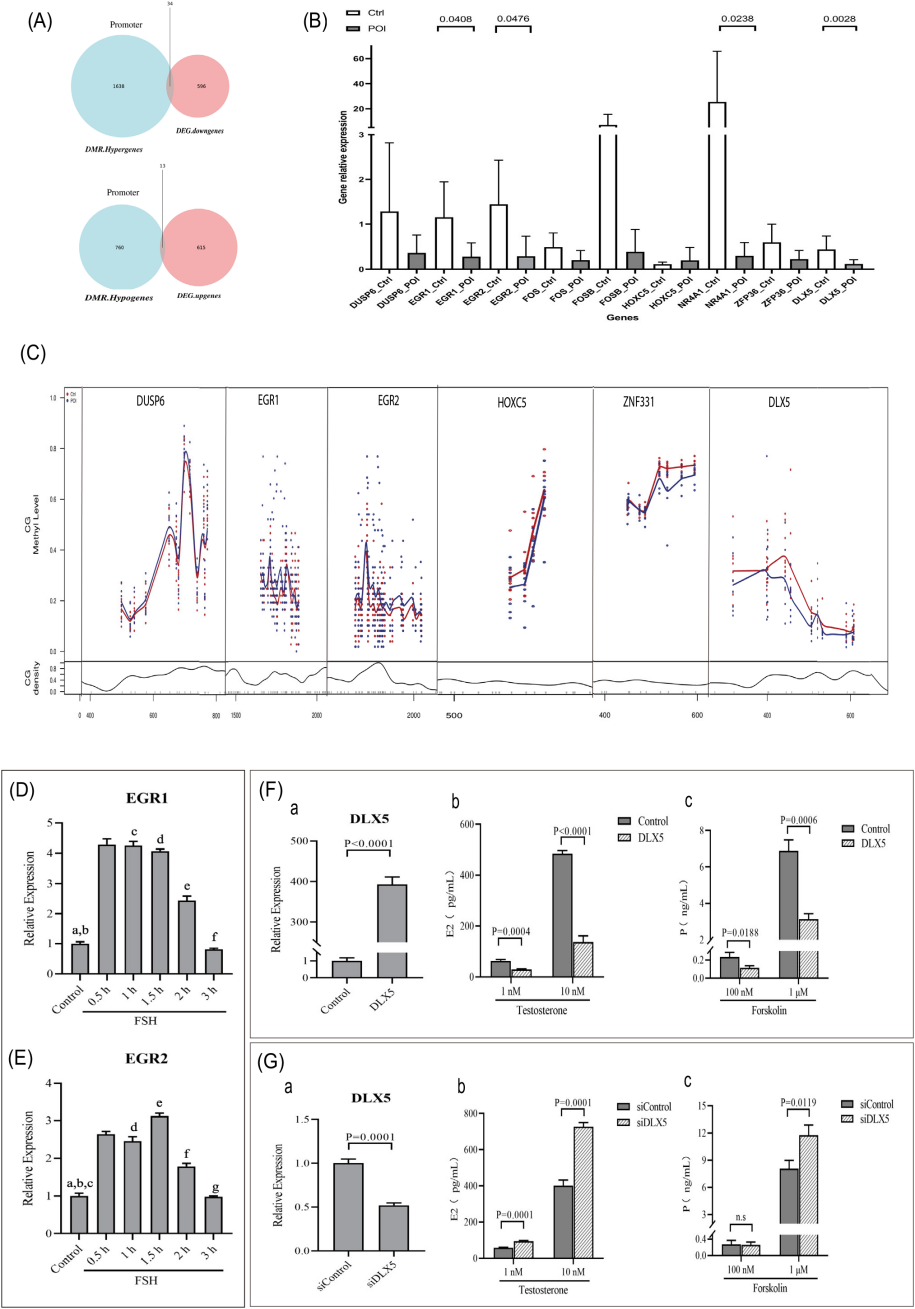
Experimental validation to detect DNA, RNA and biological function changes. (A) Venn analysis (right) on the differentially methylated region (DMR) annotation results of CG and differentially expressed gene (DEG) in the promoter region. (B) RT‐qPCR validation of the expression of nine DEGs compared with mRNA transcriptome sequencing results. (C) CG site methylation matrix to plot the methylation level change curve. The x‐axis represents the nucleotide positions in the sequence, and the y‐axis has two components: The upper part shows the methylation levels of each CG site, marked with points and fitted with a curve to illustrate the trend; the lower part displays the density change curve of the CG sites. (D) The effects of FSH treatment on the expression of EGR1 in KGN cells. (a) *p* < 0.0001, between the control group and FSH (0.5, 1, 1.5, 2 h) group; (b) *p* = 0.0093, between the control group and FSH (3 h) group; (c) No significance, between the FSH (0.5 h) group and FSH (1 h) group; (d) No significance, between the FSH (1 h) group and FSH (1.5 h) group; (e) *p* < 0.0001, between the FSH (1.5) group and FSH (2 h) group; (f) *p* < 0.0001, between the FSH (2 h) group and FSH (3 h) group. (E) The effects of FSH treatment on the expression of EGR2 in KGN cells. (a) *p* < 0.0001, between the control group and FSH (0.5, 1, 1.5 h) group; (b) *p* = 0.0002, between the control group and FSH (2 h) group. (c) No significance, between the control group and FSH (3 h) group; (d) No significance, between the FSH (0.5 h) group and FSH (1 h) group; (e) *p* = 0.0011, between the FSH (1 h) group and FSH (1.5 h) group; (f) *p* < 0.0001, between the FSH (1.5) group and FSH (2 h) group; (g) *p* < 0.0001, between the FSH (2 h) group and FSH (3 h) group. (F) The effects of overexpression DLX5 on the synthesis of Estradiol and Progesterone in KGN cells. (a) The overexpression of DLX5 in KGN cells by RT‐PCR; (b) Estradiol (E2) in DLX5 overexpressing‐KGN cells culture medium after treatment of testosterone; (c) Progesterone (P) in DLX5 overexpressing‐KGN cells culture medium after treatment of forskolin; (G) The effects of knockdown DLX5 on the synthesis of Estradiol and Progesterone in KGN cells. (a) The knockdown of DLX5 in KGN cells by RT‐PCR; (b) Estradiol (E2) in DLX5 knockdown‐KGN cells culture medium after treatment of testosterone; (c) Progesterone (P) in DLX5 knockdown‐KGN cells culture medium after treatment of forskolin.

A detailed overlap analysis was conducted between the DMRs and DEGs in the promoter region (Figure 4A). RT‐qPCR validation of the expression of nine DEGs was compared with mRNA sequencing results (Figure 4B). The upregulation or downregulation of these nine genes (GSTM1, DUSP6, MT1A, ITGB2, DLX5, ZNF331, EGR1, EGR2 and HOXC5) is consistent with the expression of the transcriptome. The amplicon bisulfite sequencing (AmpBS) was used to validate the methylation levels of target genes (DUSP6, DLX5, ZNF331, EGR1, EGR2 and HOXC5). The methylation levels of CG sites of these genes at various effective sequencing depths for each sample were statistically analysed, as shown in the Table S6. For visualisation of methylation levels at CG sites (Figure 4C), we found that EGR1, EGR2, ZNF331 and DLX5 showed a significant visual difference between the two groups. Furthermore, we performed in vitro cell experiments on select candidate genes of interest (EGR1, EGR2 and DLX5), which preliminarily confirmed their involvement in ovarian steroid hormone synthesis (Figure 4D–G).

### 3.7 The Relationship Between Methylation/Hydroxymethylation Level and Gene Expression

**FIGURE 5:**
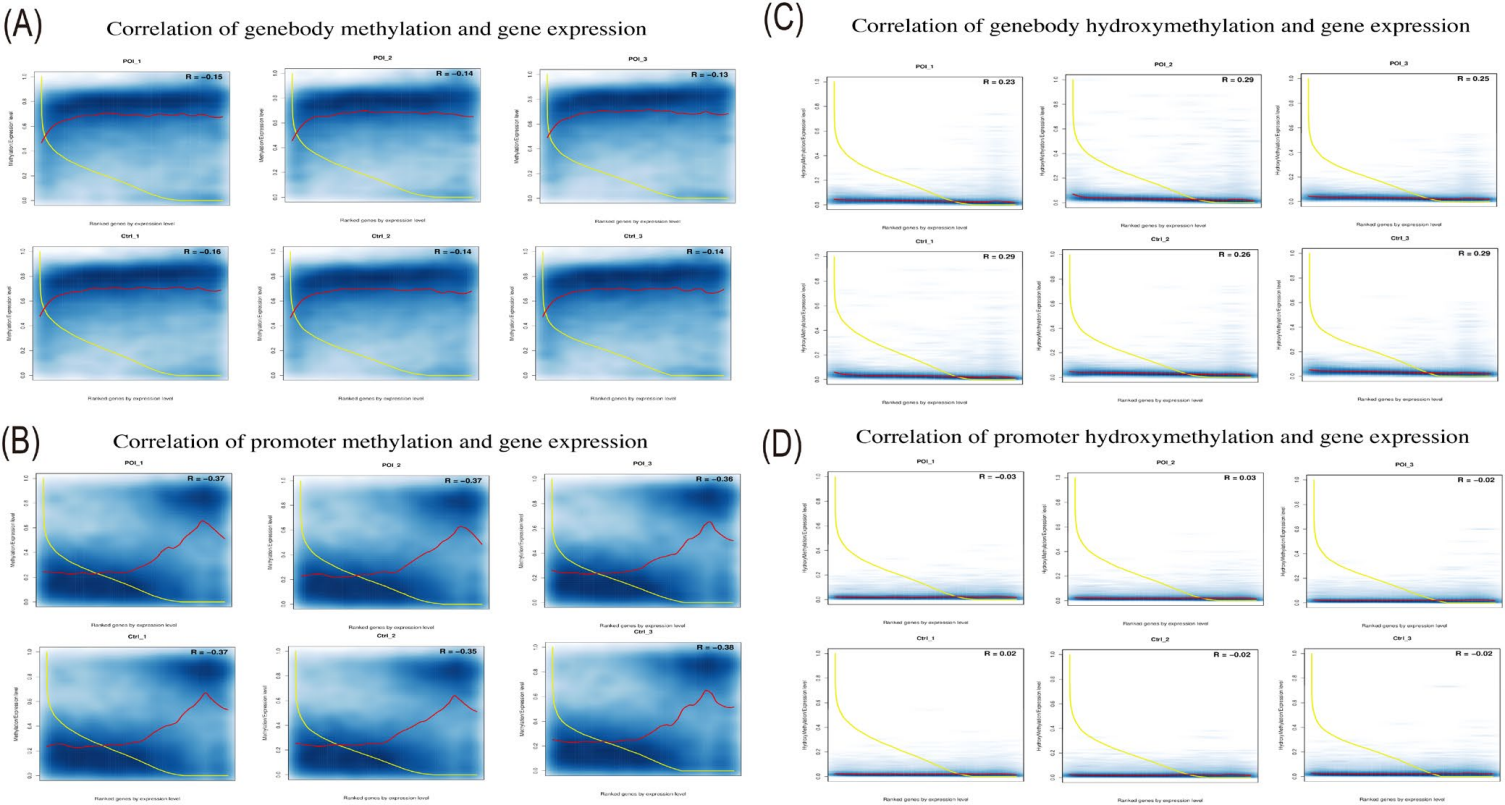
Analysis of the correlation of whole gene methylation/hydroxymethylation and gene expression. This graph visualises the correlation between the genes expression level and methylation level (A, B)/hydroxymethylation (C, D). The horizontal axis represents the rank coordinate of the gene expression levels of all genes sorted in descending order from high to low, and the vertical axis represents the methylation/hydroxymethylation level or relative expression level of genes. The scatter density plot (with a blue background) represents the distribution of all gene methylation/hydroxymethylation levels. The yellow line represents the data line for relative expression levels and the red line represents the fitting line for methylation/hydroxymethylation levels. R represents the correlation between methylation levels and relative expression levels.

An integrated analysis combining WGBS/ACE sequencing with RNA sequencing data was performed to explore the link between methylation/hydroxymethylation and gene expression. Figure 5 shows that gene body methylation is inversely related to gene expression, with most highly expressed genes having lower methylation levels (Figure 5A). Promoter methylation exhibited a bimodal distribution, affecting gene expression differently: lower methylation levels corresponded to higher expression, while higher methylation levels led to reduced expression (Figure 5B). However, both gene body and promoter regions showed minimal levels of hydroxymethylation, and no clear correlation between hydroxymethylation levels and gene expression was observed across the genome (Figure 5C,D). Additionally, we conducted a detailed overlap analysis between the DMRs and DEGs in the genebody and promoter region. The overlapping genes are provided in Table S7.

### 3.8 Methylation and POI Causative Genes, Ovarian Function Genes and Epigenetic Age Clock

**FIGURE 6:**
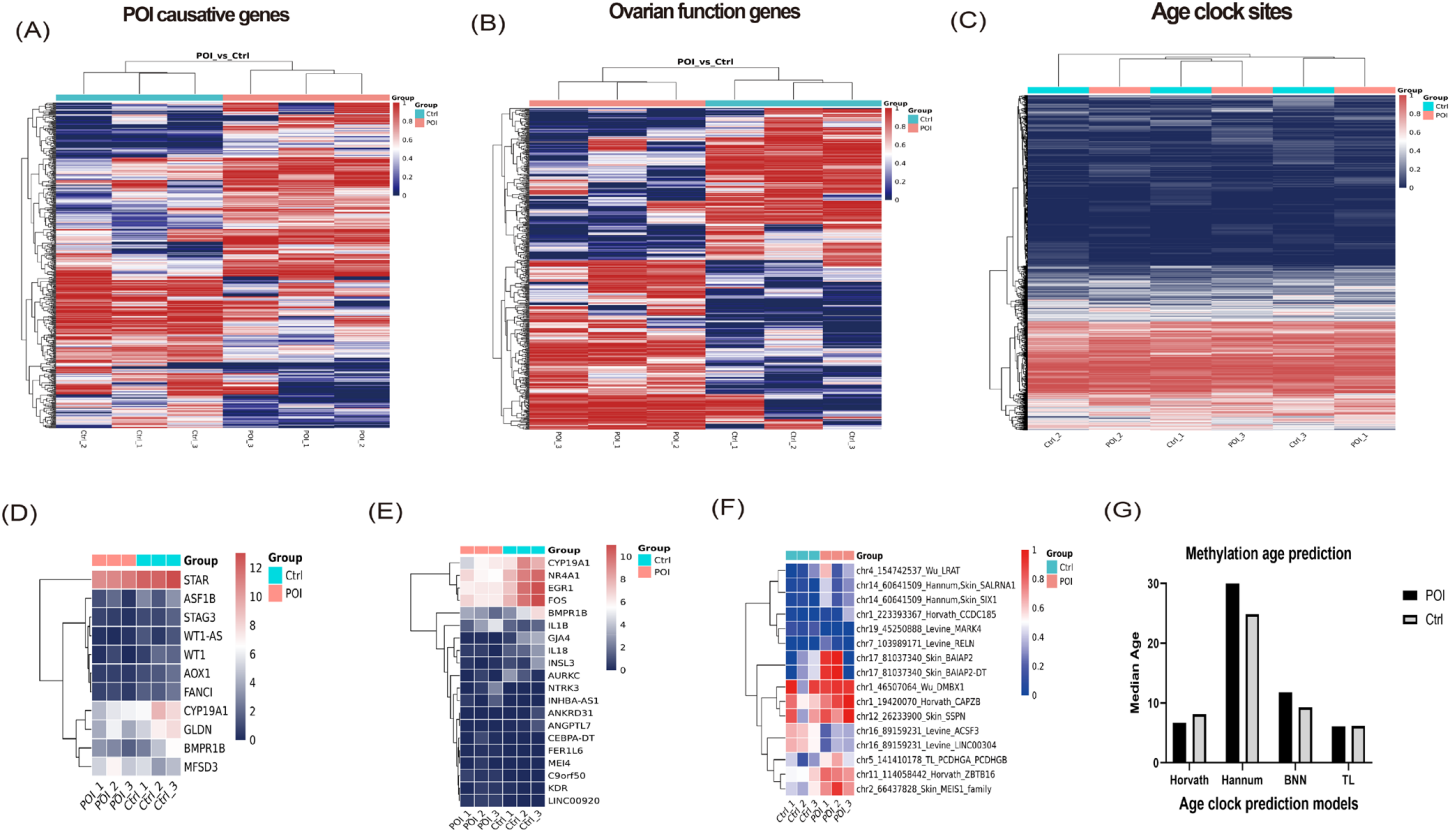
Cluster heatmap to visualise methylation levels and transcriptomic expression levels of the differentially methylated genes among the POI causative genes (A, D), ovarian function genes (B, E) and age clock CpG sites (C, F). (G) Four methylation age prediction models were employed, including the Horvath model, Hannum model, Bayesian Neural Network (BNN) model, and Telomere Length Clock (TL), to estimate the methylation age of the samples.

We have conducted additional analyses on the methylation changes among POI causative genes, ovarian function‐related genes and the epigenetic age clock (Figure 6). We firstly identified the significant differentially methylated CpG sites exhibiting POI causative/ovarian function/age clock genes. Subsequently, we generated a heatmap to visualise the methylation expression levels of these selected genes (Figure 6A–C). Additionally, we conducted an analysis of the transcriptomic expression levels of the differentially methylated genes and illustrated the findings in a separate heatmap (Figure 6D–F). The CpG sites with statistically significant differences between the two groups were identified in the methylated sites associated with the age clock (Figure 6C). The specific details of the CpG sites and related genes exhibiting significant statistical differences are provided in Table S8. We utilised four previously reported methylation age prediction models to analyse the methylation data obtained from this study and estimate the methylation age of the samples (Figure 6G). Among these models, Hannum's model predicted a methylation age that was closest to the actual age, revealing that the median methylation age of the POI group was higher than that of the control group. However, due to the small sample size, no significant differences were detected.

## 4 Discussion

This study presents the first base‐level analysis of DNA methylation and hydroxymethylation across the whole genome in human cumulus cells from POI patients, offering new insights into epigenetic modifications in POI. Key findings include (1) higher global methylation and hydroxymethylation levels in POI cumulus cells compared to healthy women; (2) a predominance of hypermethylated and hyperhydroxymethylated regions across the genome, with methylation in gene bodies negatively correlating with gene expression, especially in promoter regions; (3) further validation experiments confirmed that genes with significant differences in methylation across these omics also exhibit notable changes in their transcriptomes, and preliminary in vitro cell culture experiments indicate that these genes (specifically EGR1, EGR2 and DLX5) are involved in the synthesis of ovarian steroid hormones; (4) these identified epigenetic modifications are associated with genes implicated in POI, ovarian function and the epigenetic age clock.

### 4.1 Materials and Tissues for POI Epigenetic Research

Previous studies on ovarian aging utilised whole ovarian tissue, which may not accurately reflect the state of the oocyte due to the tissue's heterogeneity, including the medulla and connective tissue stroma [26]. Recognising that epigenetic changes in oocytes can influence early embryo and stem cell alterations, impacting gene expression across all somatic cells and organs, our study focused on the granulosa cells surrounding the oocytes. These cells, particularly cumulus cells that closely interact with the oocyte via gap junctions for metabolic and signalling molecule exchange, offer a noninvasive window into oocyte health and performance [27, 28, 29]. Unlike studies that extracted granulosa cells from follicular fluids [30, 31], which include a mix of cell types and therefore display heterogeneity (e.g., blood cells and epithelial cells) [32], our research isolated cumulus cells from cumulus cell‐oocyte complexes (COCs). This approach aims to provide a clearer understanding of the epigenetic changes occurring in oocytes, potentially explaining discrepancies with previous findings that reported no significant DNA methylation and hydroxymethylation changes with age [26].

### 4.2 DNA Methylation and Hydroxymethylation of POI

In women with POI, cumulus cells exhibit higher levels of genome‐wide DNA methylation compared to controls. This elevated methylation, particularly at gene promoters, can suppress gene expression, affecting processes critical to ovarian health‐like cell apoptosis. For instance, the downregulation of PVT1, a long noncoding RNA involved in regulating the cell cycle and apoptosis, in POI cases has been associated with its promoter's hypermethylation [33]. Experiments have shown that such downregulation of PVT1 due to hypermethylation can lead to increased apoptosis in granulosa cells by interacting with Foxo3a [30]. Similarly, increased methylation levels of autophagy‐related genes have been shown to decrease their mRNA and protein expression, contributing to the decline in ovarian function with age [34]. These findings suggest that abnormal DNA methylation in cumulus cells may play a significant role in the deterioration of ovarian function seen in POI.

Granulosa cells experience significant DNA demethylation during follicle development and ovulation, particularly after treatments like equine chorionic gonadotropin (eCG), affecting genes related to luteinization and LH targets [35]. Our study collected granulosa cells 36 h post human chorionic gonadotropin injection, aligning with a phase of LH‐induced demethylation. Despite this demethylation phase, granulosa cells from the POI group exhibited higher methylation levels than those from the control group. This suggests that even after LH‐induced demethylation, the higher methylation status in the POI group could interfere with critical processes like oocyte maturation and ovulation. This is supported by our findings of decreased oocyte quality and downregulation of the chromosome segregation pathway in the POI group. Our study underscores the importance of examining epigenetic changes in cumulus cells during late follicular development to uncover potential causes of POI.

### 4.3 Validation of Target Genes

Early growth response (EGR) protein is one of the target genes that interests us. It is a transcription regulator that exerts its function through binding to the promoter of target genes to regulate their transcription activity [36]. It has been reported that EGR1/2 expression can be upregulated by gonadotropin in granulosa cells, which is related to follicle maturation and ovulation. Further, the EGR1 increased in granulosa cells and promotes the progression of ovarian hyperstimulation syndrome [37], which are contrary to the characteristics of ovarian dysfunction (poor ovarian response). This suggested that EGR1/2 involves the ovarian response to gonadotropin in the process of ovarian stimulation, which has been confirmed in our cellular experiments (Figure 4D,E).

DLX5 gene encodes a member of a homeobox transcription factor gene family and is thought to be an important regulator of bone formation [38]. A few studies reported that upregulation of DLX5 promotes ovarian cancer cell proliferation by enhancing IRS‐2‐AKT signalling [39]. In our experiment, we found a significant increase in the methylation levels of DLX5 in granulosa cells of patients with POI, particularly in the promoter region. At the same time, the mRNA expression of DLX5 was downregulated. There is limited research on DLX5 in ovarian granulosa cells, and this study provides preliminary evidence that DLX5 may be involved in the steroid hormone synthesis process in ovarian granulosa cells (Figure 4F,G). Our subsequent research will continue to explore the molecular biology processes of DLX5 involvement in POI in granulosa cells.

### 4.4 DNA Methylation and Hydroxymethylation in Promoter and Genebody

Our findings confirm the classical understanding that DNA methylation in gene promoters generally leads to gene silencing by preventing transcription initiation [40, 41]. We observed a bimodal distribution in our data (Figure 5B), with some genes showing low methylation and others high methylation levels. Consistently, gene expression in both POI and control samples was inversely related to DNA methylation at promoter sites, aligning with the notion that increased methylation suppresses gene expression. However, exceptions were noted, including 18 upregulated genes with hyper promoter methylation and 19 downregulated genes with hypo promoter methylation (Table S7), indicating that not all gene expressions strictly follow this pattern.

Our research indicates that gene body methylation plays a significant role in regulating gene expression across various tissues, contrary to the traditional focus on promoter methylation. We observed that high gene expression levels often corresponded with low levels of gene body methylation. This suggests a complex relationship between gene expression and methylation patterns, extending beyond the promoter regions to include 5′‐UTR and 3′‐UTR areas adjacent to the gene body [42, 43]. Notably, studies, including our own, have identified strong associations between gene expression changes and methylation near the transcription end site (TES), particularly in genes like AMH that are crucial for ovarian function [44]. These findings highlight the importance of considering the entire genomic context, including hypomethylated gene bodies and promoters, to fully understand the epigenetic mechanisms underlying POI pathology.

### 4.5 Methylation and POI Causative Genes, Ovarian Function Genes and Epigenetic Age Clock

Our analysis identified significant methylation differences in genes traditionally associated with POI pathology or ovarian function when comparing the POI group to the control group. Concurrently, the transcriptomic expression levels of these DEGs exhibited significant variations. Based on these findings, we hypothesise that the potential factors related to POI may influence the methylation levels of certain ovarian function genes, and changes in methylation levels indirectly lead to alterations in DNA transcription expression, ultimately resulting in the potential development of POI.

Given the ovary's early functional decline with age [45, 46], we analysed methylation levels at epigenetic clock sites (blood clock) in POI and control groups, presenting our findings in a heatmap. Among 1447 sites examined, 17 showed significant methylation differences (listed in Table S8), though no overall significant changes were detected (with over 98.5% of sites showing no difference). By four previously methylation age prediction models to estimate the methylation age of the samples, the predicted methylation age of POI samples may show an upward trend than controls. This aligns with studies suggesting that the epigenetic age of cumulus and mural granulosa cells does not mirror chronological age [47, 48], indicating a unique epigenetic timeline within the ovarian compartment [49].

## 5 Conclusion

Our research findings indicate that POI in human cumulus cells is characterised by widespread DNA hypermethylation and hyperhydroxymethylation throughout the genome, suggesting a potential pathophysiological mechanism. Increased levels of hypermethylation in promoters and gene bodies may influence gene expression and function, potentially contributing to the development of POI. The correlation between hyper/hypomethylation and gene expression regulation in this study offers valuable insights for identifying potential epigenetic biomarkers and treatment targets for POI. Preliminary experimental validation was conducted to ascertain the involvement of candidate genes, specifically EGR1, EGR2 and DLX5, in the synthesis of ovarian steroid hormones. Notably, our results suggest a significant association between these epigenetic modifications and genes related to POI, ovarian function and the epigenetic age clock. Future investigations should focus on elucidating the specific molecular mechanisms underlying hypermethylation, as well as identifying the genes and pathways that are influenced by these modifications.

## Author Contributions

**Wenhao Shi:** conceptualization (equal), data curation (equal), funding acquisition (lead), writing – original draft (lead), writing – review and editing (equal). **Dongyang Wang:** investigation (equal), validation (equal). **Xia Xue:** investigation (equal), resources (equal). **Sen Qiao:** investigation (equal), methodology (equal). **Wei Zhang:** writing – original draft (supporting), writing – review and editing (supporting). **Juanzi Shi:** resources (equal), supervision (equal). **Chen Huang:** conceptualization (equal), visualization (equal).

## Ethics Statement

The animal study was reviewed and approved by the Ethics Committee of Northwest Women's and Children's Hospital (2023‐057) and complied with all relevant ethical regulations. Written informed consent was obtained from all participants in this study. All experimental methods abided by the Helsinki Declaration.

## Conflicts of Interest

The authors declare no conflicts of interest.

## Supporting information


**Figure S1.** Cumulative coverage of the three types of 5mC methylation modes based on effective sequencing depth. The horizontal coordinate represents the cumulative sequencing depth, and the vertical coordinate represents the corresponding coverage. The three main 5mC methylation modes are identified with different colours.
**Figure S2.** The methylation/hydroxymethylation distributed on chromosomes between POI and control samples (A/B).
**Figure S3.** The circos diagrams display the overall differentially methylated region/differentially hydroxymethylated region (A) and hyper/hypo region (B) across the entire genome.
**Figure S4.** GO and KEGG pathway analysis. (A) GO analysis for promoter‐hyper/hypo of DMR and DhMR; (B) KEGG pathway analysis for promoter‐hyper/hypo of DMR and DhMR. The total number of selected genes within the GO pathways on the dot plot is shown in brackets. The colour of the dot indicates the adjusted *p*‐value (*p* < 0.05 and FDR < 0.05), and the size of the dot is proportional to the number of DEGs in the given pathway.

**Table S1.** Patients' characteristics.
**Table S2.** Sequencing quality control.
**Table S3.** qPCR primers.
**Table S4.** List of POI causative genes and ovarian function genes.
**Table S5.** CpG sites of Age clock.
**Table S6.** Validation CpG sites.
**Table S7.** Genes ID DMR&DEG.
**Table S8.** CpG sites of WGBS and age clock.

## Data Availability

The datasets used and/or analysed during the current study are available from the corresponding author on reasonable request. The data that support the results of current study is available on Gene Expression Omnibus websites (GSE267640, GSE267641, GSE267643).
