# Supplementary material for: Epigenomic Landscape of Human Cumulus Cells in Premature Ovarian Insufficiency Using Single‐Base Resolution Methylome and Hydroxymethylome
Source: J Cell Mol Med. 2024 Dec 20;28(24):e70284. doi: 10.1111/jcmm.70284 (PMC11661916; doi:10.1111/jcmm.70284)
Supplement: Supplementary file 1 — Figure S1. Cumulative coverage of the three types of 5mC methylation modes based on effective sequencing depth. The horizontal coordinate represents the cumulative sequencing depth, and the vertical coordinate represents the corresponding coverage. The three main 5mC methylation modes are identified with different colours. Figure S2. The methylation/hydroxymethylation distributed on chromosomes between POI and control samples (A/B). Figure S3. The circos diagrams display the overall differentially methylated region/differentially hydroxymethylated region (A) and hyper/hypo region (B) across the entire genome. Figure S4. GO and KEGG pathway analysis. (A) GO analysis for promoter‐hyper/hypo of DMR and DhMR; (B) KEGG pathway analysis for promoter‐hyper/hypo of DMR and DhMR. The total number of selected genes within the GO pathways on the dot plot is shown in brackets. The colour of the dot indicates the adjusted p‐value (p < 0.05 and FDR < 0.05), and the size of the dot is proportional to the number of DEGs in the given pathway. [file JCMM-28-e70284-s001.pdf]

Appendices

Supplementary Figures

The file includes:

Fig. S1-S4

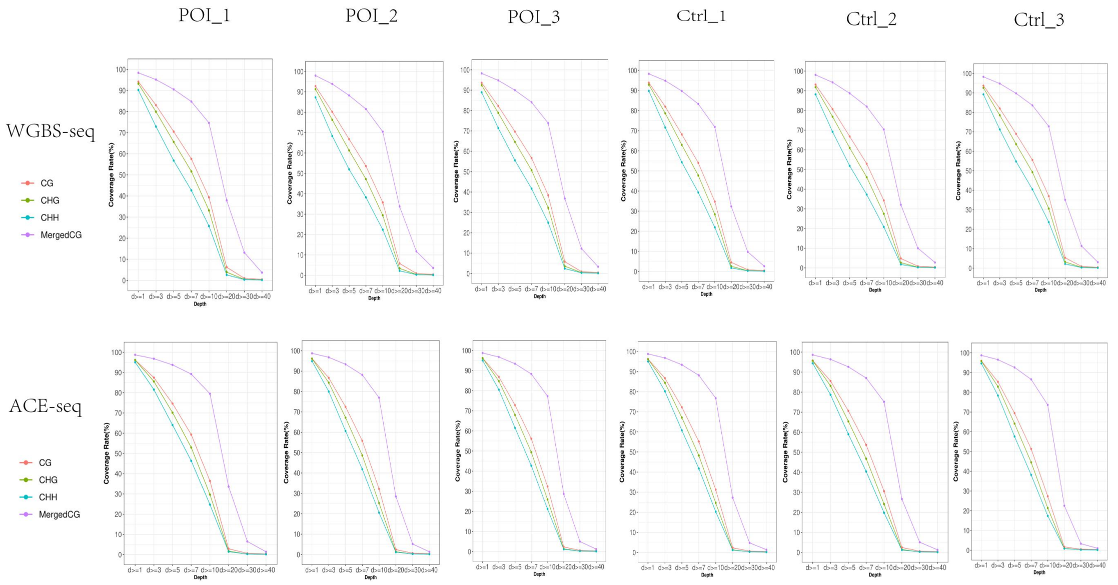

Figure S1 Cumulative coverage of the three types of 5mC methylation modes based on effective sequencing depth. The horizontal coordinate represents the cumulative sequencing depth, and the vertical coordinate represents the corresponding coverage. The three main 5mC methylation modes are identified with different colors.

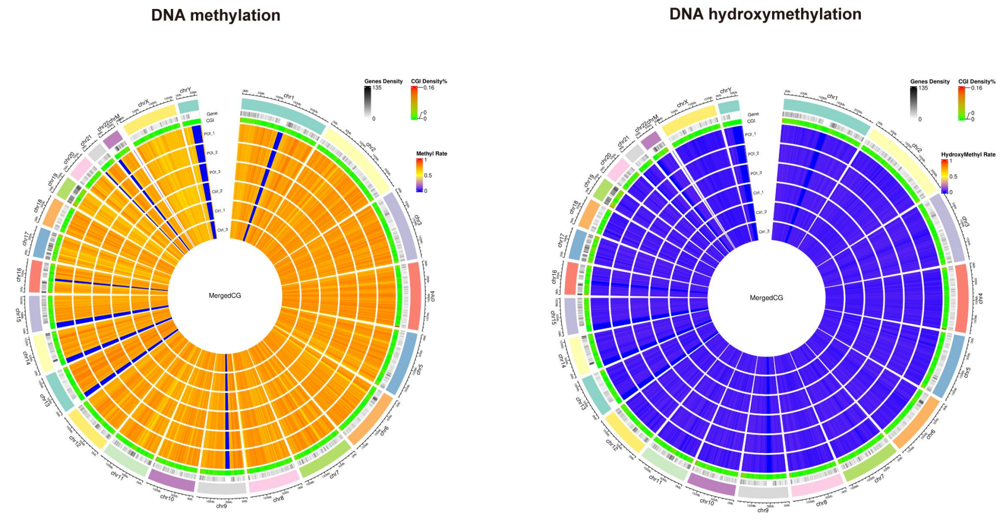

Figure S2 The methylation/hydroxymethylation distributed on chromosomes between POI and control samples (A/B).

Differentially methylated regions **Differentially hydroxymethylated regions**

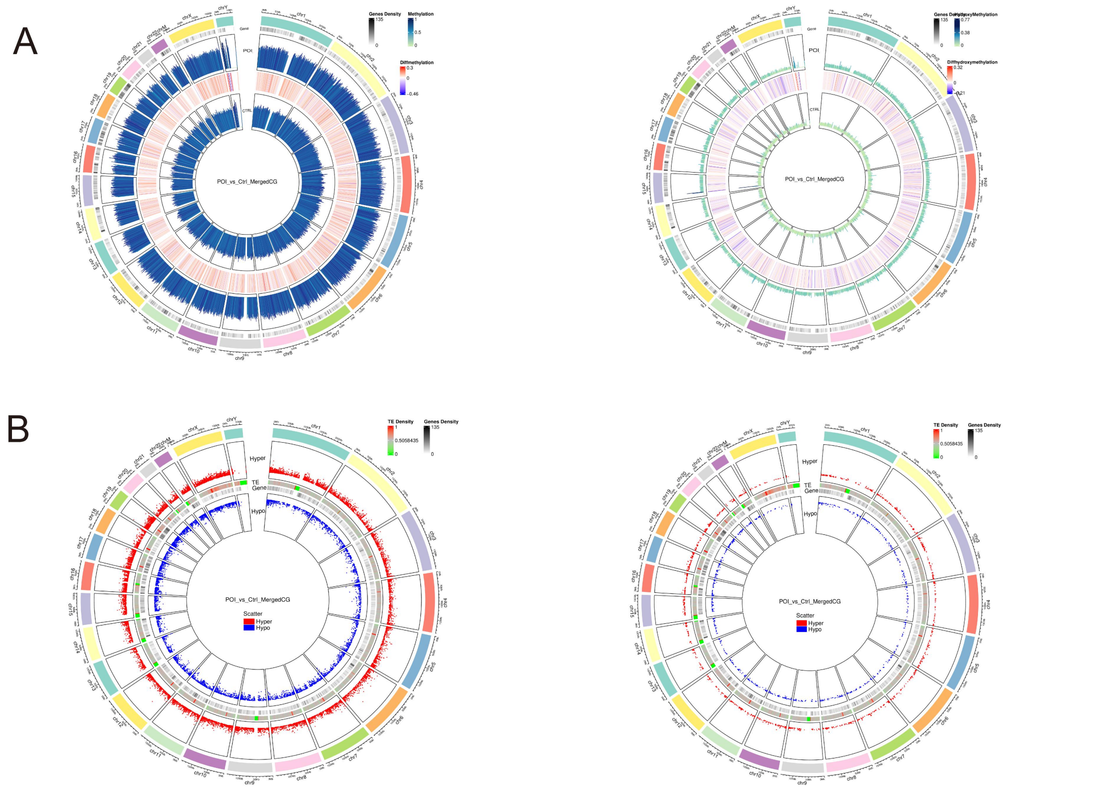

Figure S3 The circos diagrams display the overall differentially methylated region/differentially hydroxymethylated region (A) and hyper/hypo region (B) across the entire genome .

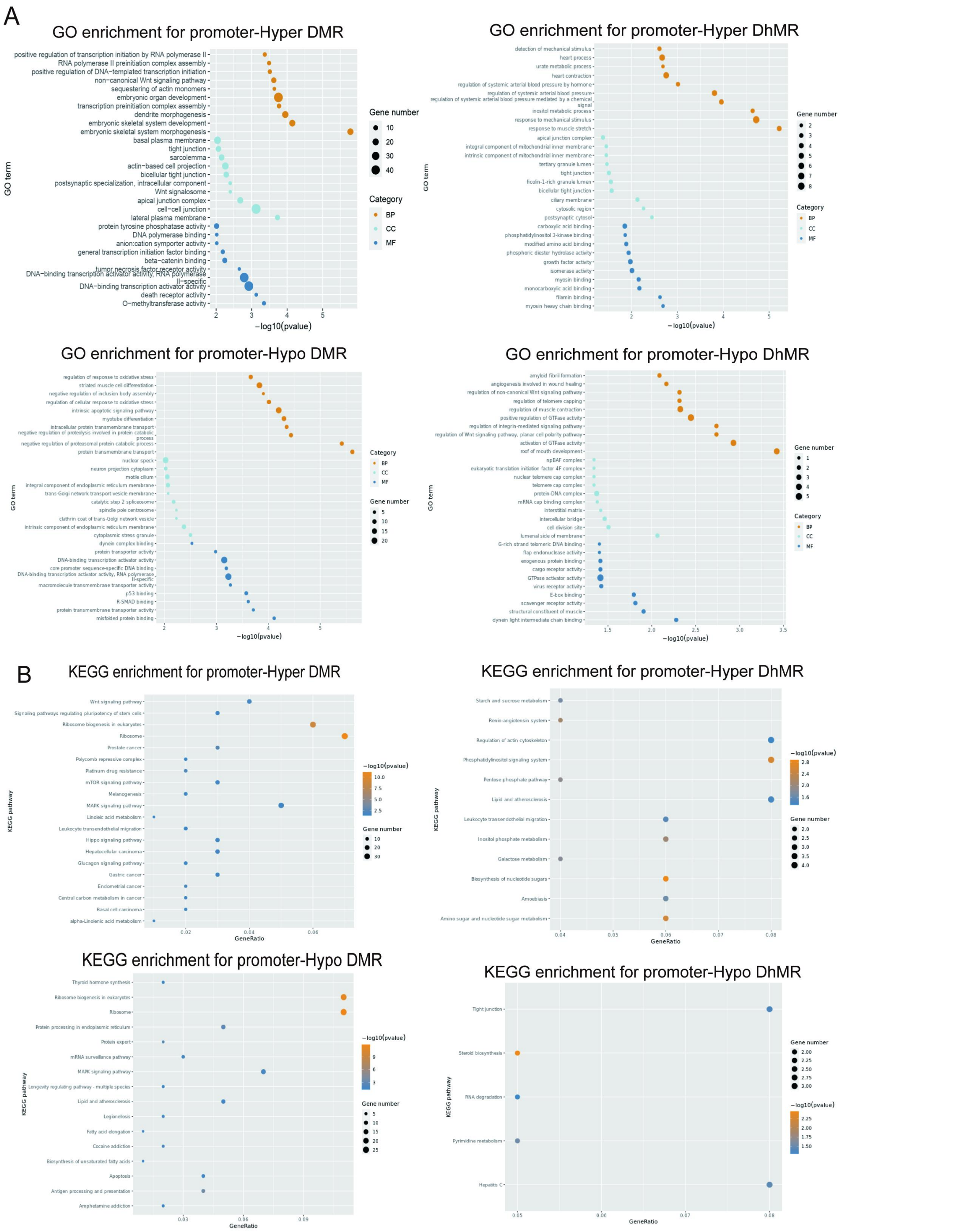

Figure S4 GO and KEGG pathway analysis. (A) GO analysis for promoter-hyper/hypo of DMR and DhMR; (B) KEGG pathway analysis for promoter-hyper/hypo of DMR and DhMR. The total number of selected genes within the GO pathways on the dot plot is shown in brackets. The color of the dot indicates the adjusted p-value ($p < 0.05$ and FDR < 0.05), and the size of the dot is proportional to the number of DEGs in the given pathway
